# Anti-biofilm peptides can rescue fluconazole and amphotericin B efficacies against *Candida albicans*

**DOI:** 10.1038/s41598-025-10315-4

**Published:** 2025-07-09

**Authors:** Ann-Kathrin Kissmann, Vanessa Mildenberger, Markus Krämer, Daniel Alpízar-Pedraza, Ernesto M. Martell-Huguet, Julio A. Perez-Erviti, Ahmet Cetinkaya, Joanna Pietrasik, Anselmo J. Otero-Gonzalez, Carolina Firacative, Armando Rodríguez, Ludger Ständker, Tanja Weil, Steffen Stenger, Frank Rosenau

**Affiliations:** 1 Institute of Pharmaceutical Biotechnology, Ulm University, Albert-Einstein- Allee 11, 89081 Ulm, Germany; 2 Max Planck Institute for Polymer Research Mainz, Ackermannweg 10, 55128 Mainz, Germany; 3 Center for Pharmaceutical Research and Development (CIDEM), 26th Avenue, No. 1605, Nuevo Vedado, La Habana, 10400 Cuba; 4 Center for Protein Studies, Faculty of Biology, University of Havana, 25 and I, La Habana, 10400 Cuba; 5 Institute of Polymer and Dye Technology, Lodz University of Technology, Stefanowskiego 16, Lodz, 90-537 Poland; 6 Studies in Translational Microbiology and Emerging Diseases (MICROS) Research Group, School of Medicine and Health Sciences, Universidad del Rosario, Bogota, 111221 Colombia; 7 Core Facility for Functional Peptidomics, Faculty of Medicine, Ulm Peptide Pharmaceuticals (U- PEP), Ulm University, 89081 Ulm, Germany; 8 Core Unit of Mass Spectrometry and Proteomics, Faculty of Medicine, Ulm University, 89081 Ulm, Germany; 9 Institute for Medical Microbiology and Hygiene, University Hospital Ulm, 89081 Ulm, Germany

**Keywords:** Antimicrobial peptides, Amphotericin B, *Candida albicans*, Fluconazole, Resistance, Fungal infection, Antifungal agents, Biofilms

## Abstract

*Candida albicans* infections are a global health thread and challenge healthcare environments due to acquired resistances against prominent antifungals like amphotericin B and fluconazole, which additionally have severe adverse effects. The peptide Pom-1 originally isolated from the freshwater mollusk *Pomacea poeyana*, and its derivatives Pom-1 A-F have proven their potential against biofilms of clinical *C. albicans* isolates and were suspected to act without candidolytic pore-formation. Here, Pom-1 and its derivatives were shown to act as neutralizing antimicrobial peptides (nAMPs) inhibiting cell-cell interactions and hence biofilm formation. Combining Pom-1 nAMPs with fluconazole and amphotericin B restored their efficacy against resistant *C. albicans* isolates. Addition of Pom-1 nAMPs allowed to reduce required concentrations to 10–50% below their described effective therapeutic doses. This opens doors not only to mitigate adverse effects of fluconazole and amphotericin B therapies, but also towards novel combination therapies against *C. albicans* as a severe re-emerging pathogen.

**Supplementary Information:**

The online version contains supplementary material available at 10.1038/s41598-025-10315-4.

## Introduction

Systemic infections by pathogenic yeasts, especially by *Candida albicans*, have been highlighted already in 2018 as one of the most common bloodstream infections worldwide and in the United States^1^. In this time, candidemia affected on average approximately 9 per 100,000 individuals, with the number of cases varying with the population and the geographic region the patients live in. In 2017 the Centers for Disease Control and Prevention (CDC) estimated the burden of candidemia for the US health system and suspected that approximately 25,000 cases of this invasive infection occur nationwide each year^2^. Based on CDC’s surveillance data it has been suggested that the in-hospital all-cause (crude) mortality among people with candidemia is up to 25%^3,4^. In addition, an alarming number of 75% of all women are confronted with a fungal infection by species of the pathogenic genus *Candida*, including *Candida parapsilosis*, *C. glabrata*, or *C. tropicalis*, during their lifetime, with 5–8% of adult women developing recurrent vulvovaginal candidiasis, which is defined as four or more episodes every year^5,6^. In 85–95% of cases, however, these infections are in fact caused by *C. albicans*^7^. Effective drugs exist to treat fungal infections with echinocandins, azoles, and polyenes, as well as established examples^8,9^. Among them fluconazole, as a fungistatic agent, represents the first-line treatment option for diseases like endophthalmitis, meningitis, for infections of oral or vaginal mucosa^10^, whereas amphotericin B with its great antifungal/fungicidal activity is favored for severe systemic or invasive infections^11^.

In addition to the sheer infection numbers, prophylactic prescriptions and resulting overuse of classical medications have forced the development of different resistance mechanisms, hence qualifying candidiasis as an infection of truly re-emerging severity and a dramatically increasing problem for healthcare systems^4,10,12–14^. Moreover, a severe limitation for administration of amphotericin B has been summarized to reside in its (infusion-related) toxicity, divided into acute and chronic toxicity resulting in nausea, vomiting, rigors, fever, hypertension or hypotension, and hypoxia and chronic nephrotoxicity as a consequence of different renal injury mechanisms^15^. Although during fluconazole treatment, intestinal indisposition and skin affection have been observed as side effects for more than 1% of the volunteers in an early clinical study, with additionally occurring occasional hepatic, hematologic and renal abnormalities, these abnormalities were considered low compared to other systemically administered drugs like amphotericin B^16^. Nevertheless, a potential therapeutic option allowing a significant reduction of the needed dose for such classical antimycotics appears to be generally desirable.

**Fig. 1:**
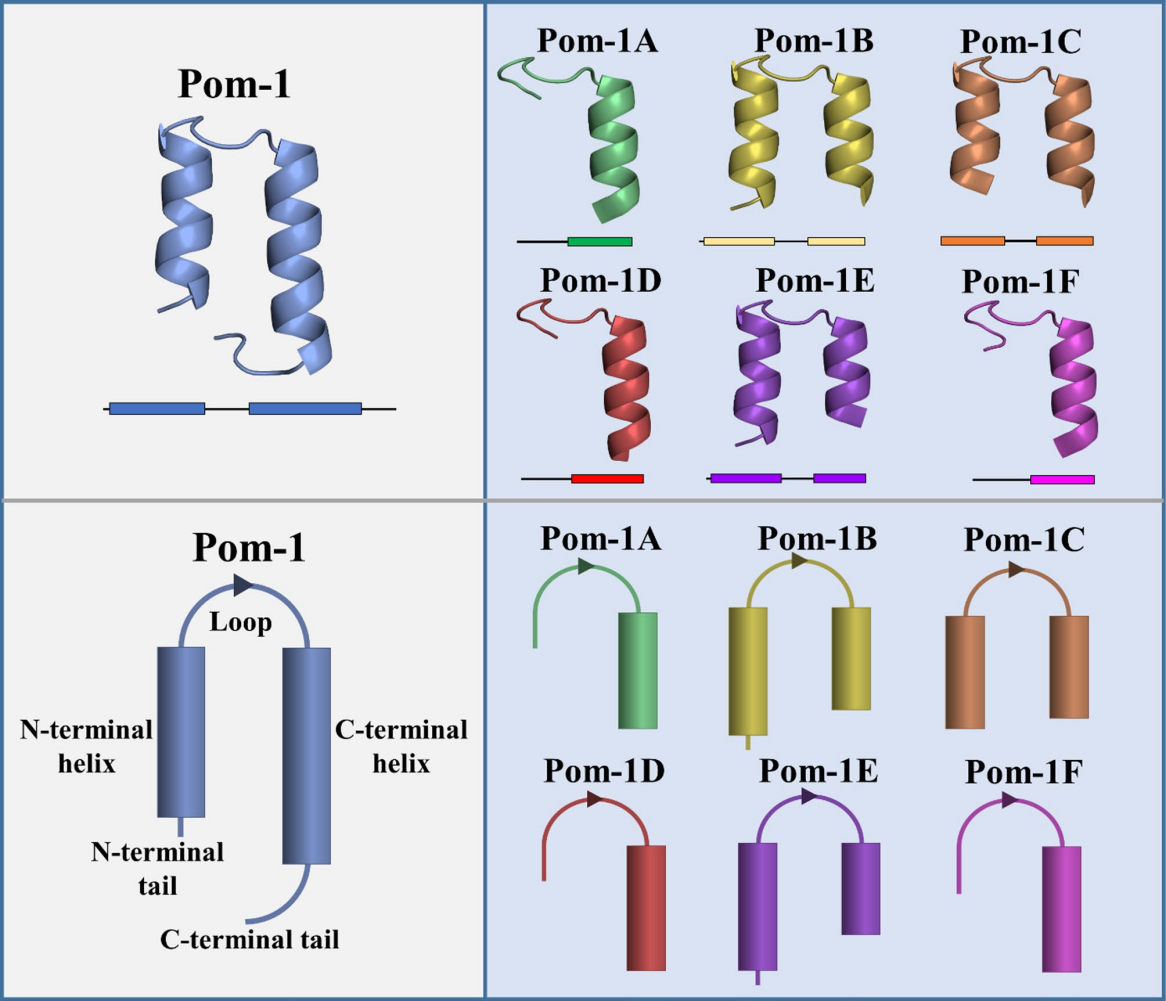
Derivatives of the naturally occurring *Pomacea poeyana* lead peptide known as Pom-1. Depiction of the original molecule Pom-1 and its six derivatives Pom-1 A to Pom-1 F represented as ribbon models with QUARK and SwissModel and schematic representation of the α-helical structures.

A potent class of drug molecules with a wide range of activity against various pathogens includes fungi, are natural and synthetic antimicrobial peptides (AMPs)^17–19^. Conventional AMPs have two major principle modes of action and can either be membrane-active by integrating the peptide into the fungal membrane thereby forming different types of pores^20,21^, or they exert intracellular effects like the inhibition of key metabolic pathways^22,23^. A “classical” AMP is membrane-active and the functional and structural integrity of the cell membrane is disturbed during its action by pores that are formed in the pathogenic membrane, thereby simply affecting the viability of cells. Diverse mechanistic models have been developed to describe the productive binding and membrane integration events of AMPs to bio-membranes^24,25^. Mollusks have proven their enormous potential as biological resources for the identification of potent AMPs as their natural anti-infective protection concept exclusively relies on an innate immune system involving an arsenal of AMPs^26,27^. We have introduced peptides from this class of organisms, which were first isolated from the Cuban freshwater snail *Pomacea poeyana* and then chemically resynthesized. These Pom-1 and Pom-2 peptides and their derivatives had antimicrobial activity not only against the different bacteria, but also against biofilm formation of various *Candida* species, and in addition, were characterized by a remarkably low cytotoxicity against human cells^28,29^. Based on their pronounced dedication towards biofilm inhibition, Pom-1 derivatives (Fig. 1) and other snail-derived^28,30–32^ peptides have been discussed to possess a different mechanism of action, which rather involves a simple binding of surface epitopes thereby shielding those structures from exerting their physiological roles in cell-cell or cell-substrate contacts than classical pore formation^28,29,33,34^.

**Fig. 2:**
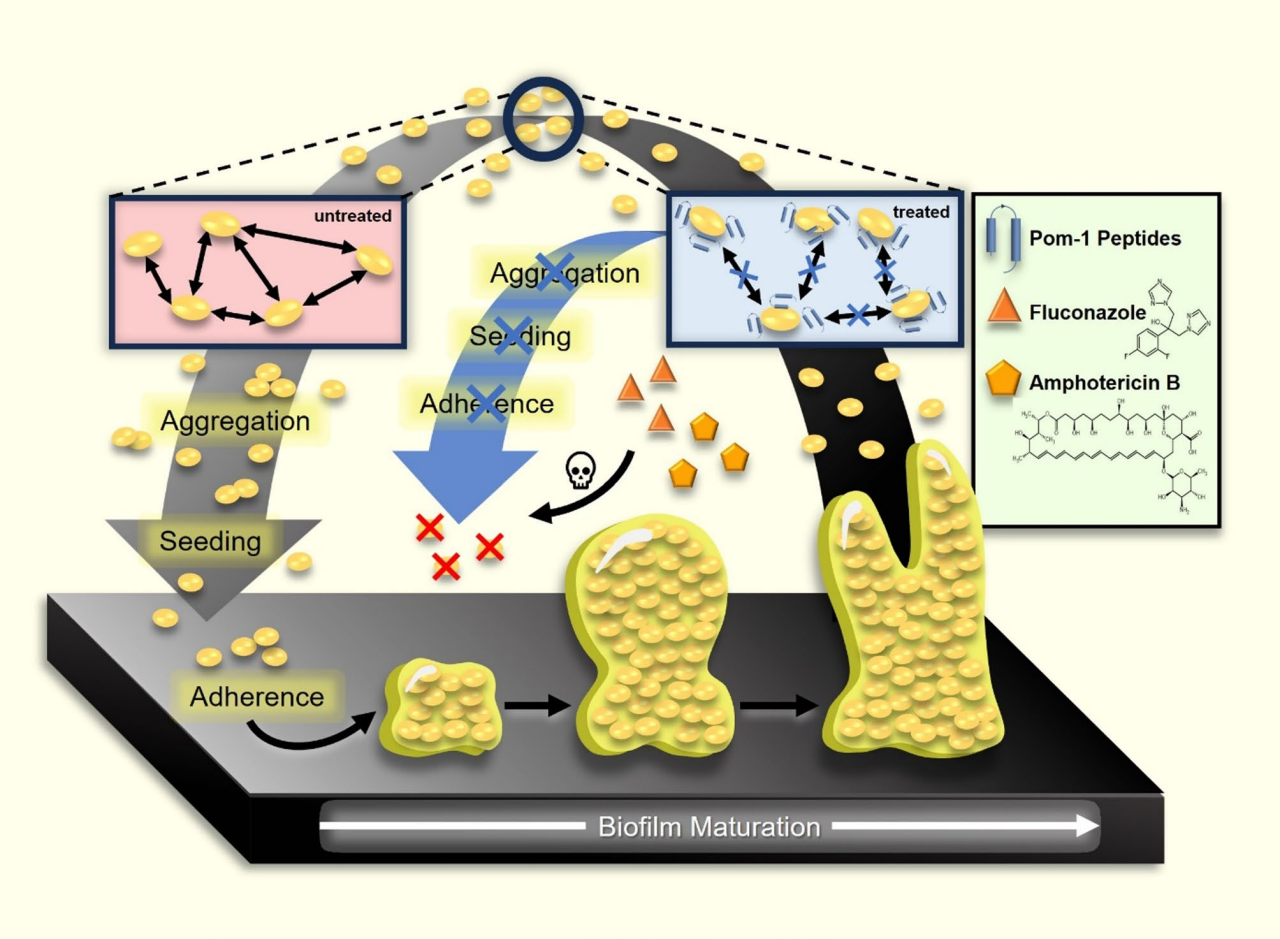
Schematic illustration of proposed Pom-1 peptides mode of action with enhanced susceptibility of Pom-1 treated yeast cells to the classical antifungals amphotericin B and fluconazole. Planktonic yeast cells that are not subjected to Pom-1 treatment form aggregates (“aggregation”) and are therefore less vulnerable to antifungals amphotericin B and fluconazole and are able to attach to surfaces and form biofilms. *C. albicans* planktonic cells that are treated with Pom-1 are not able to aggregate and therefore last longer in the planktonic phase, which leads to them being more susceptible for treatment with fluconazole and amphotericin B and consequent killing of the cells and prevention of biofilm formation.

Here, we show that Pom-1 and its derivatives have aggregation-inhibiting activities of *C. albicans* cells in the planktonic phase (Fig. 2). A permeabilization assay measuring the uptake of fluorescent marker molecules revealed that, compared to a known classical AMP, the Pom-1 peptides do not exhibit pore-forming abilities. Moreover, we show that the Pom-1 derivatives either as individual peptides or finally as mixtures of all six peptides in combination with fluconazole or amphotericin B, drastically increased the efficacy of the traditional antifungals resulting in the total killing of 96% of clinical isolates from invasive infections, including strains resistant against one of the drugs or both. The required doses of fluconazole and amphotericin B could be reduced by 50% compared to the traditional concentrations used in *C. albicans* therapy. This demonstrates that their combination with Pom-1 peptides could help to restore the efficacy of antifungal drugs that would otherwise be ineffective due to pathogen resistance.

## Results

The peptides Pom-1 and its derivatives Pom-1 A-F have previously been described as being not directly cytotoxic and deadly for *C. albicans* cells in the planktonic phase and have therefore been discussed as probably not belonging to the class of pore-forming AMPs^33,34^. However, the failure to form pores in *Candida* cell membranes has so far never been verified experimentally. Classical pore-forming AMPs possess the intrinsic capability to establish interactions with membrane phospholipids which then initiate a cascade of molecular events leading to productive immersion and final generation of a permeable pore.

**Fig. 3:**
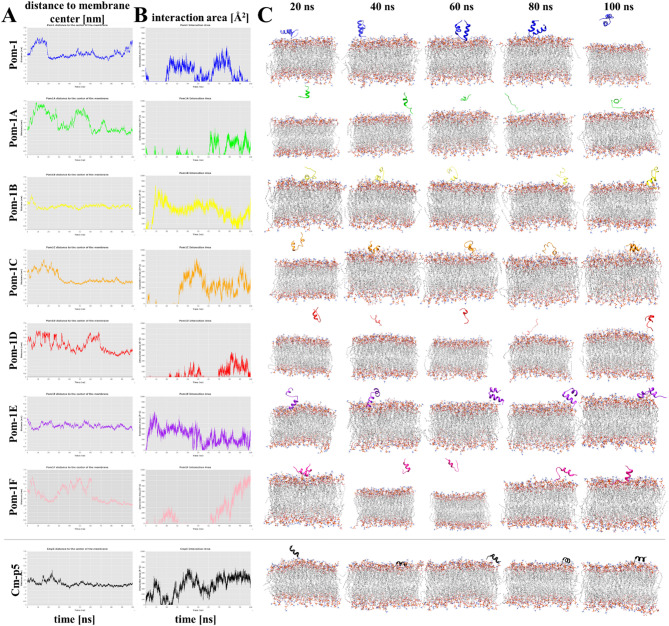
Molecular dynamic simulations of interactions of Pom-1 and derivatives with *C. albicans* model phospholipid biomembranes. The analysis pipeline was based on the CHARMM-GUI input generator and the NAMD 2.14 package with the CHARMM36 force field. (**A**) Distance of the peptides (Pom-1 and Pom-1 A-F) to the center of the membrane simulated for an interval of 0–100 ns. The peptide Cm-p5 as a known membrane-permeabilizing molecule served as a positive control. (**B**) Respective surface area of the peptides interacting with the membrane. (**C**) Position of the peptides (represented in cartoon) at 20, 40, 60, 80 and 100 ns of the molecular dynamic simulations, membrane phospholipids are shown in gray with red head groups. In A, B and C the same color scheme was used for the peptides with blue, green, yellow, orange, red, purple, pink for Pom-1, Pom-1 A, Pom-1B, Pom-1 C, Pom-1D, Pom-1E, Pom-1 F and black for Cm-p5, respectively.

In general, Pom-1 and its derivatives showed low affinity for *C. albicans* membranes (Fig. 3). As it can be observed in Fig. 3A, the peptides did not bind stably to the membrane, and there was a relatively wide fluctuation in the distance between the protein and the membrane. Some peptides remain close to the membrane as Pom-1B, Pom-1 C, Pom-1E, and at the last 20 ns Pom-1 F. However, these peptides also present high fluctuations in the surface interaction area. Although short-term interactions of the peptides with the membrane appeared to occur, the interactions were characterized by instant dissociation indicating their presumably extremely weak stability (Fig. 3B). In consequence, the analysis of the peptide positions at 20, 40, 60, 80, and 100 ns, revealed that all the Pom-1 derivative peptides as well as the superordinate wild type Pom-1 do not adopt a stable conformation over the membrane and hence no pore-forming capability (Fig. 3C).

**Fig. 4:**
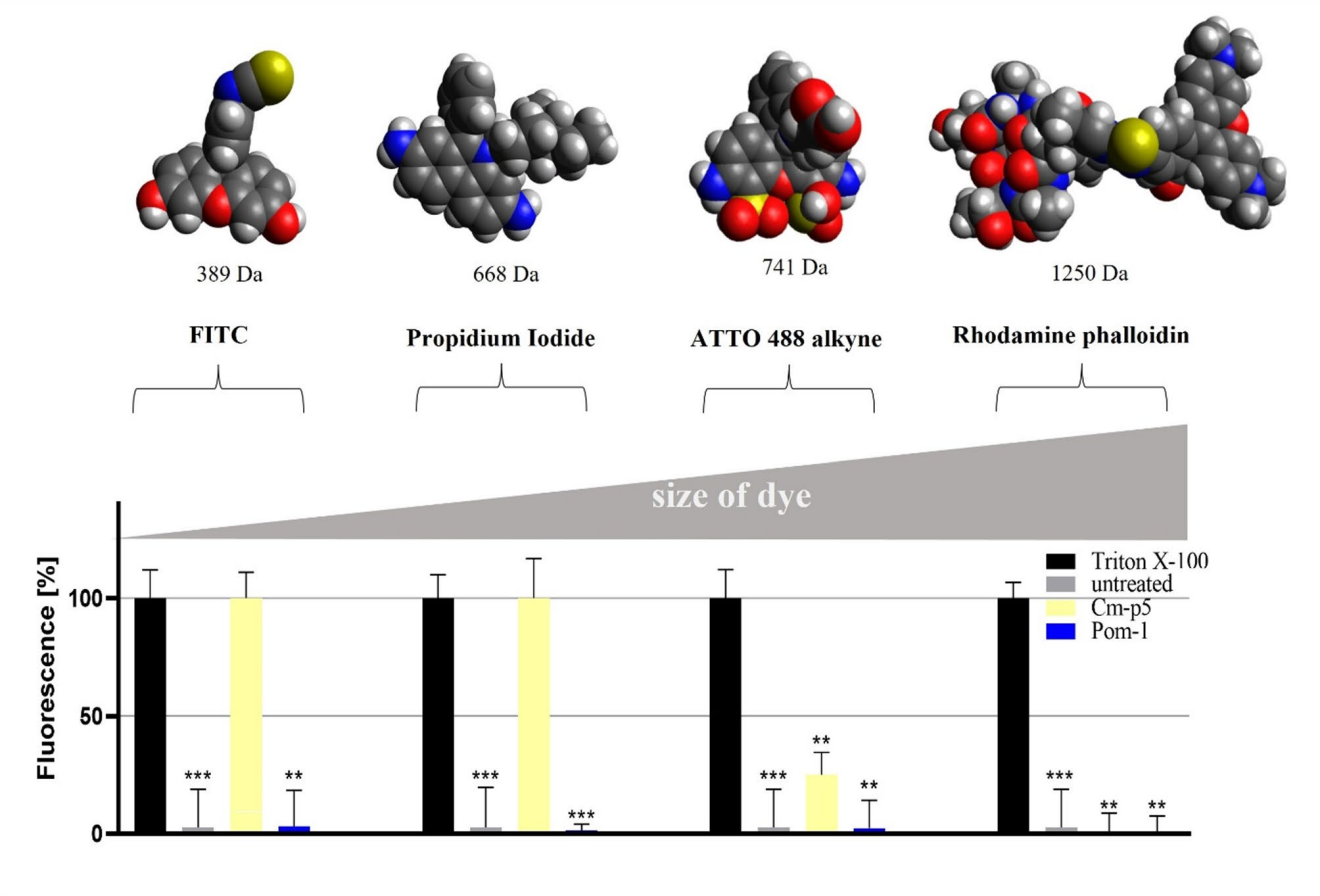
Influence of Pom-1 on cell permeability. Depicted is the permeability of *C. albicans* cells treated with the known membrane-disruptive peptide Cm-p5, the detergent Triton X-100 and the original Pom-1 peptide. Structural representations of the fluorescent dyes FITC, Propidium Iodide, ATTO 488 alkyne and Rhodamine phalloidin are shown as well as their molecular sizes. Treated cells were stained with these dyes and the fluorescence signal was detected and represented as bars. All experiments were performed in triplicate, and the error bars depict the standard deviations. Statistical analysis was performed with a *t*-test; *p*-values < 0.05 were considered significant (*p < 0.05; **p < 0.01; ***p < 0.001). The columns without specific labeling show no significant differences (ns).

Based on the molecular modeling in silico results, we wanted to verify this finding particularly with the superordinate wild type Pom-1 as the relevant lead structure and applied a membrane permeabilization assay based on fluorescent dyes of increasing molecular size to evaluate pore formation in the presence of the known antimicrobial peptide Cm-p5, which is known for its membrane disruptive activity^31,32,35^ as a peptide positive control and Triton X-100 as a physicochemical detergent positive control. Triton X-100 permeabilized *Candida* cells for all dyes and Cm-p5 was permissive only for FITC, propidium iodide and partly for ATTO 488 alkyne but excluded the largest dye rhodamine phalloidin, whereas the original Pom-1 peptide completely failed to permeabilize the *Candida* cells (Fig. 4).

**Fig. 5:**
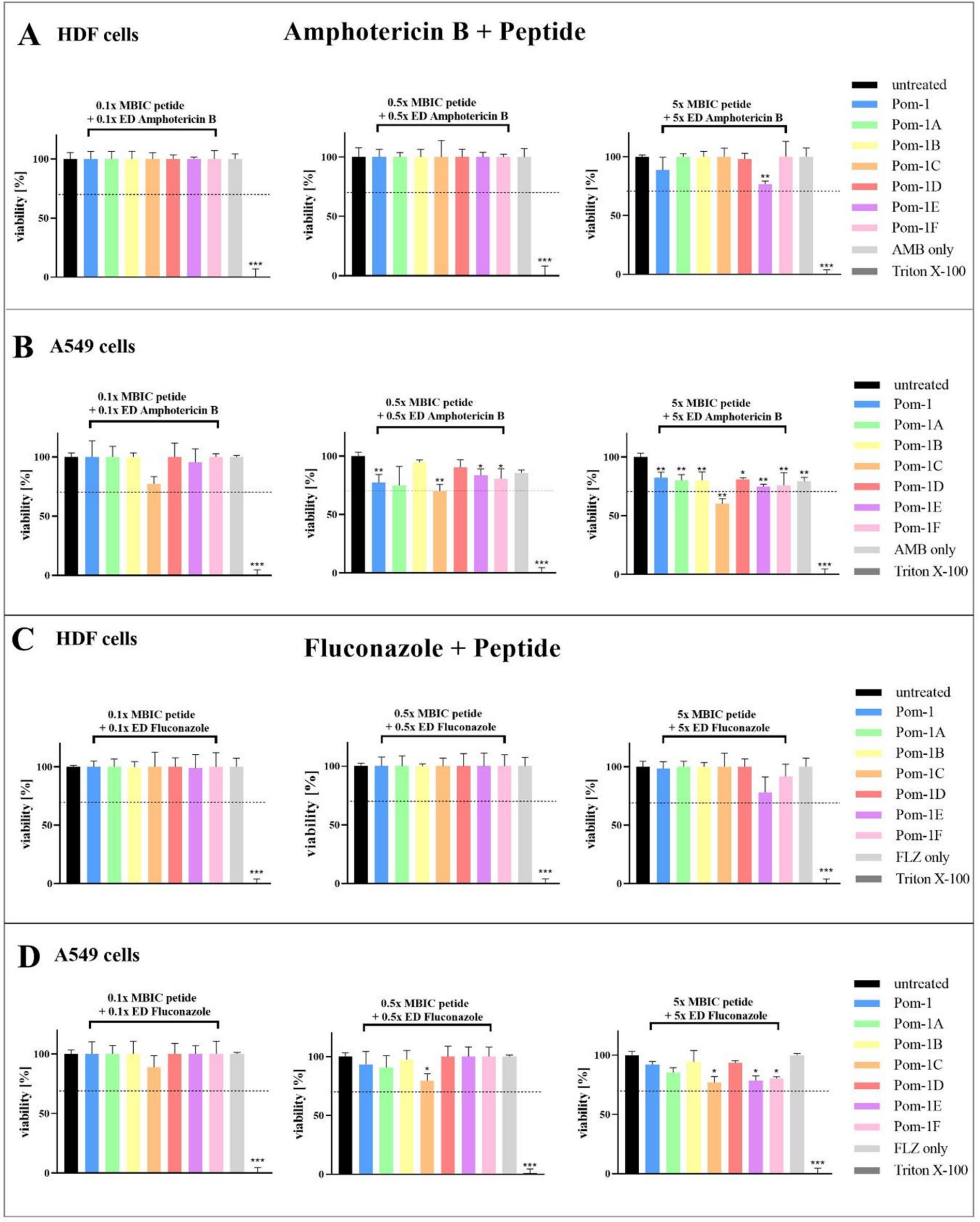
Effects of the Pom-1 derivatives in combination with amphotericin B (AMB) and fluconazole (FLZ) on the viability of HDF and A549 cell lines. (**A**) Cell viability of HDF cells after addition of 0.1x, 0.5x and 5x MBIC peptide + ED amphotericin B. (**B**) Viability of A549 cells after treatment with 0.1x, 0.5x and 1x MBIC peptide + ED amphotericin B. (**C**) Cell viability of HDF cells after addition of 0.1x, 0.5x and 5x MBIC peptide + ED fluconazole. (**D**) Viability of A549 cells after treatment with 0.1x, 0.5x and 5x MBIC peptide and ED fluconazole. Triton X-100 was included as a control. All the experiments were performed in triplicate, and the error bars depict the standard deviations. Statistical analysis was performed with a *t*-test; *p*-values < 0.05 were considered significant (*p < 0.05; **p < 0.01; ***p < 0.001). The columns without specific labeling show no significant differences (ns). A cell viability lower than 70% was considered cytotoxic and is marked as a dashed line.

Their simple physical mode of action to form harmful pores in bio-membranes is generally a severe drawback of classical AMPs and the major reason for considerable cytotoxicity also for human cells^36^. Fortunately, Pom-1 and its derivatives have been proven to be non-toxic for human cells, which was the original reason for us to postulate a mode of action different from simple pore-forming^28,33,34^. A reasonable pharmaceutical use of synergistic effects of combinations of the classical anti-*Candida* drugs fluconazole and amphotericin B with the Pom-1 derivative peptides strictly requires that the cytotoxicity, as one of the major criteria for potential combination therapy, does not increase and exceed a tolerable threshold, which is generally accepted as ≥ 70% viability of the remaining cells, according to the EN ISO 10993-5 guideline for biological evaluation of medical devices^37^. The benchmark in our study were final concentrations of fluconazole and amphotericin B at maxima of 8 µg/mL and 2 µg/mL, respectively. These thresholds represent the accepted maximal therapeutic concentrations (i.e. the maximally used effective dose (ED)) in humans of these classical and worldwide broadly used antimycotics with activities against *Candida albicans*^10,11,14^. Biofilms are not only a typical type of lifestyle for *C. albicans* but also represent a crucial property of these pathogenic yeasts to express their full virulence potential. The Pom-1 derivative peptides have shown convincing and distinct activities against *C. albicans* and have been suggested as novel biofilm-dedicated drugs with minimal biofilm inhibitory concentrations (MBIC) of 2.5 µg/mL for the reference laboratory strain *C. albicans* ATCC 90,028^28,29,33,34^. In a previous study we have used a set of 27 invasive clinical isolates collected in a sampling campaign in the Ulm Hospital. We showed that using the 1x MBIC the single peptides failed to completely inhibit biofilm formation, which was also the case when 10x MBIC was applied in the experiments^33^. However, in combination, i.e. when the peptides were used in mixtures, the anti-biofilm activity was drastically enhanced^33^. Consequently, in the follow-up study presented here, we intended to evaluate whether synergistic effects can also be observed when the individual Pom-1 peptides are used in mixtures with the classical pharmaceutic antifungals amphotericin B and fluconazole. Main questions were if it was possible to reduce amphotericin B and fluconazole concentrations as they are normally applied in therapies and whether in turn the peptides can rescue their efficacies with special respect to fluconazole and/or amphotericin B resistant clinical isolates. As antifungal concentrations of interest for the evaluation of cytotoxicity caused by this possible combination therapy, we used 50% and 10% in comparison to 500% of each substance as a control benchmarking extreme concentrations with no real practical use (i.e. 0.5x, 0.1x and 5x ED). The single peptides were used each at 0.5x, 0.1x and 5x of their MBIC. Besides human dermal fibroblasts (HDF) as a self-evident cell type for testing the effects of drugs often applied topically, we again chose A549 cells. This cell line can be regarded as “hypersensitive” towards (classical) AMPs, which in turn means that if a peptide cannot kill A549 cells non-cancer cells *a fortiori* will profit from the fact that the particular peptide is non-toxic for them and will survive its presence^38^. Whereas not a single peptide in combination with the amphotericin B concentrations reduced viability below the 70% threshold for HDF cells (Fig. 5A), the combination of the peptide Pom-1 C and amphotericin B in the highest concentrations significantly reduced the viability of A549 cells to 60% (Fig. 5B). In contrast, also for A549 all other combinations proved to be non-cytotoxic in this assay even at these extreme control concentrations (Fig. 5B). For all assays the Triton X-100 detergent cell lysis controls reduced cell viabilities as expected to zero. Markedly, the combinations of peptides with fluconazole also proved to be perfectly non-toxic in every concentration applied in our assay system (Fig. 5C,D).

These initial but crucially important observations prompted us to evaluate our thesis that Pom-1 and its derivatives may generally act, via their known anti-biofilm activity, as pharmaceutical compounds neutralizing cell-cell interactions thereby reducing the intrinsic aggregation propensity of *C. albicans*, resulting in an increased maintenance of individual cells already in the planktonic phase. Subsequently, this modification or inhibition of normal aggregation behavior would be in consequence the reason for impaired biofilm formation of peptide-treated cells (Fig. 2). In this context, this would then increase the accessibility and susceptibility of those cells for the classical antifungals and in consequence qualify the peptides as sensitizers for the increase of efficacy and, in the case of resistant strains, for restoration of their impact in future therapeutic strategies.

**Fig. 6:**
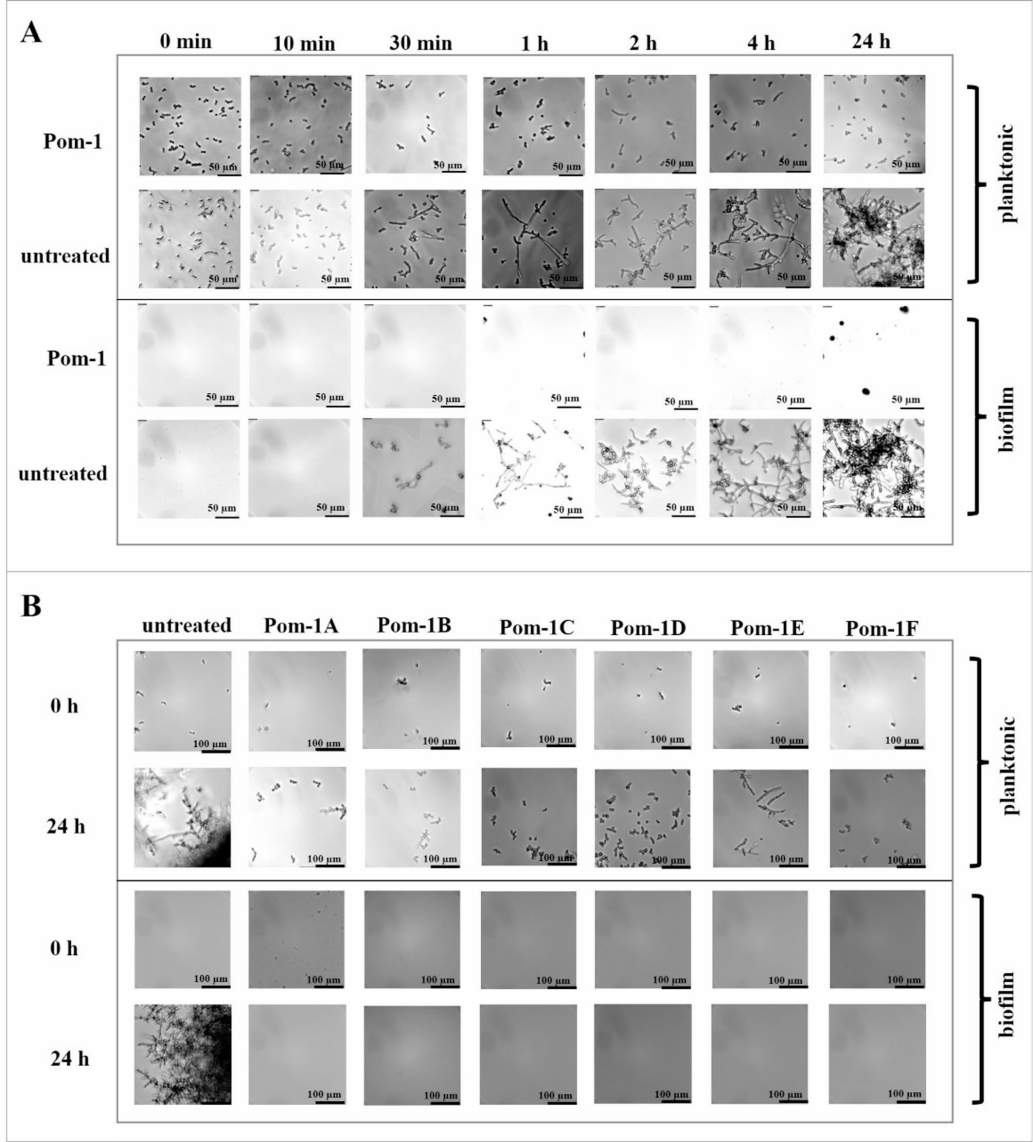
Pom-1 and its derivatives influence *C. albicans* cell morphology and prevent aggregation and subsequent biofilm formation (**A**) Planktonic *C. albicans* cells and *C. albicans* biofilm with and without treatment with Pom-1 after incubation times of 0 min, 10 min, 30 min, 1 h, 2 h, 4 h and 24 h. (**B**) Biofilm and planktonic cells of *C. albicans* with and without treatment with Pom-1 A-F after 0 h and 24 h. Microscopic analyses were conducted using a Leica DMi8 coded (Leica Microsystems CMS GmbH, Wetzlar, Germany) microscope of *C. albicans* biofilm and planktonic cells with and without treatment with Pom-1 and its derivatives.

This idea was tested with the original Pom-1 peptide as the lead peptide, which was used to monitor cell aggregation and the influence on initial early steps of biofilm formation on the solid polystyrene-based surface of a microtiter plate. Cells of the reference laboratory strain *C. albicans* ATCC90028 as a model were cultivated in this biofilm formation kinetic experiment for 24 h with sampling after 0 h, 10 min, 30 min, 1 h, 2 h, 4 h and 24 h, and addition of Pom-1 at the MBIC of 2.5 µg/mL^34^ at the beginning of cultivation (0 h). The planktonic phase was then removed from the remaining biofilm and both phases were analyzed by microscopy. After 24 h, the treated cells showed no biofilms which is in agreement with our previous findings^28^ and thus delivered the expected microscopic confirmation (Fig. 6A, “Biofilm”, upper panel), whereas untreated cells had formed regular biofilms (Fig. 6A, “Biofilm”, lower panel). In the planktonic phase (i.e. the culture supernatant), differences between treated and untreated cells became visible already after 30 min, culminating during the time course in drastic morphological alterations after 24 h. Treated cells kept their round-shaped morphology and their appearance as singular cells even at the end of the experiment (Fig. 6A, “Planktonic”, upper panel). In contrast the untreated control predominantly formed higher architectures of hyphae-like large cellular aggregates during the experiment (Fig. 6A, “Planktonic”, lower panel). For the Pom-1 derivatives Pom-1 A-F, the same analyses of planktonic cells and biofilms were performed and, consistently, the aggregation inhibitory effects of the Pom-1 derivatives were indistinguishable from the lead peptide after 24 h (Fig. 6B).

**Fig. 7:**
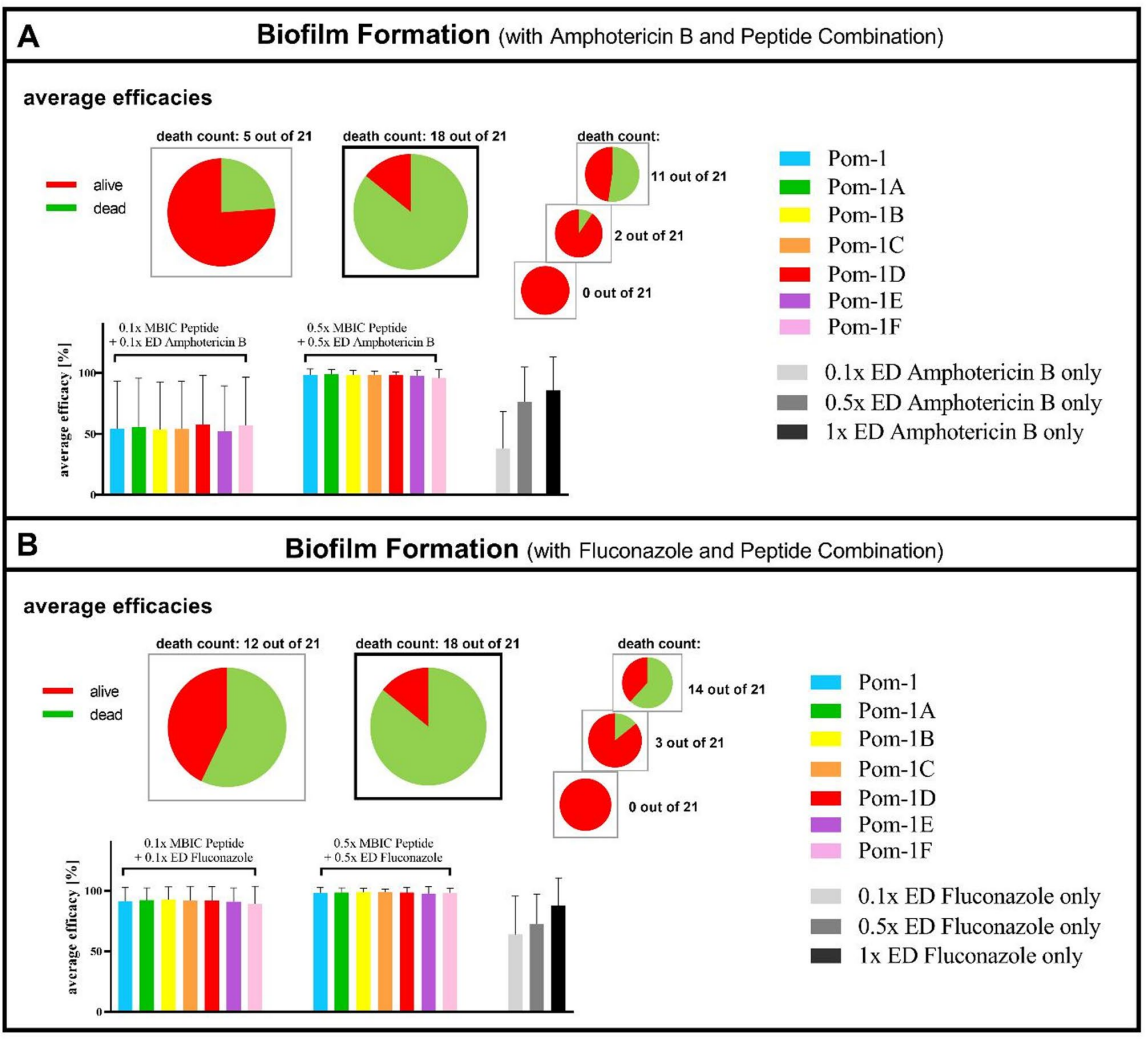
Rescued anti-biofilm effect of amphotericin B and fluconazole against *C. albicans* clinical isolates when combined with Pom-1 and its derivatives. Average efficacies of the Pom-1 derivatives (0.1x and 0.5x MBIC) in combination with (**A**) amphotericin B (0.1x and 0.5x ED) as well as amphotericin B only (0.1x, 0.5x and 1x ED) and (**B**) with 0.1x MBIC peptide and 0.1x ED fluconazole as well as 0.5x MBIC peptide and 0.5x ED fluconazole against biofilm formation. The number of *Candida* strains, for which no biofilm formation was detected, for at least one of the peptide derivatives is depicted as the “death count” and represented as pie charts. Error bars represent experiments conducted as triplicate.

To test the possibility that a combination of Pom-1 peptides and classical antimycotics may be more efficient than the established drugs alone and to evaluate whether the peptides may be suitable to increase fluconazole or amphotericin B efficacies thereby opening the option to reduce their required effective dose for the sake of the intended reduction of generally harmful side effects, three different concentrations of peptides and antifungals were used in the biofilm assay (“0.1x level”: 0.1x MBIC peptides + 0.1x ED antifungal; “0.5x level”: 0.5x MBIC peptides + 0.5x ED antifungal; “1.0x level”:1x ED antifungal as a reference). In this biofilm assay the 1x level amphotericin B achieved a complete inhibition of biofilms with 11 of 21 for 52% of the strains (Figs. 7A and S2A) (“death count” and the skull pictogram in figures S2 and S3). Compared to this the 0.1x level of peptides and antifungal reached inhibition ratio of 24% and the 0.5x level already resulted in a complete biofilm inhibition of 86% (18 of 21), which in turn indicates an improvement of 45% of these strains. amphotericin B alone at a concentration representing 0.5x ED in contrast allowed a complete biofilm inhibition for only 2 of 21 strains (9.5%) and 0.1x ED totally failed in biofilm inhibition for all strains. For the second traditional antifungal fluconazole the results were similar with also 18 of 21 strains inhibited in biofilm formation for the most potent combination (0.5x level) with the remarkable finding that the 0.1x level of peptides and fluconazole already inhibited 12 of 21 isolates (Figs. 7B and S2B).

**Fig. 8:**
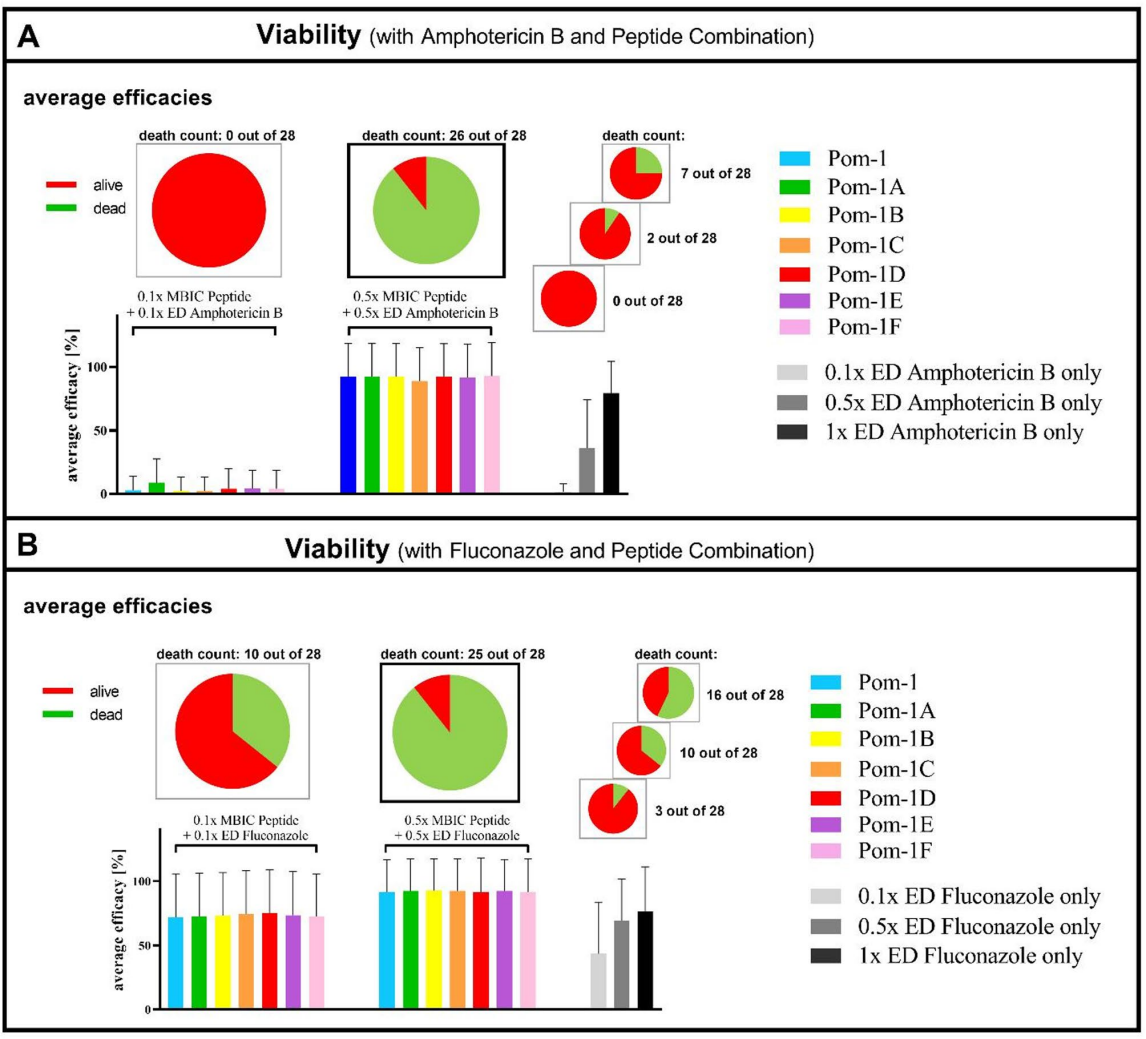
Enhanced efficacy against planktonic cell viability of amphotericin B and fluconazole when combined with Pom-1 and its derivatives. Average efficacies of the Pom-1 derivatives (0.1x and 0.5x MBIC) in combination with (**A**) amphotericin B (0.1x and 0.5x ED) as well as amphotericin B only (0.1x, 0.5x and 1x ED) and (**B**) with 0.1x MBIC peptide and 0.1x ED fluconazole as well as 0.5x MBIC peptide and 0.5x ED fluconazole against planktonic cells. The number of *Candida* strains, for which no metabolic activity was detected, for at least one of the peptide derivatives is depicted as the “death count” and represented as pie charts. Error bars represent experiments conducted as triplicate.

The assessment of antifungal efficacies was done by comparison of the activities of amphotericin B and fluconazole either, alone or combination with the Pom-1 peptides against *C. albicans* cells in the planktonic lifestyle. The aim of the combination treatment was the reduction of fungal cultures to zero viability at the lowest concentrations possible. Compared to the traditional effective dose of amphotericin B (1x ED), which was fully active against only 25% of the isolates (7 of 28), the 0.1x level failed completely and was thus not an improvement to the pure antifungal (0.1x ED). In contrast, the 0.5x level represented a drastic improvement because in this scenario 93% (26 of 28) of the strains were killed completely, whereas the pure antifungal control at the same 0.5x ED alone only accomplished a 7% reduction (Fig. 8A and S3A). Remarkably, all of the known amphotericin B resistant isolates^33^ were among the strains completely eradicated in the presence of peptides and the 0.5x ED of amphotericin B (Fig. 8A), a result, which prompted us to interpret the peptide effect as a rescuing of the previously lost killing ability of the antimycotic. The rescuing effect of the peptides was similar in combination with fluconazole resulting in a severereduction of fungal metabolic activity for 89% (25 of 28) isolates at the 0.5x level. Here, the 0.1x level allowed a direct judgement of the rescuing effect because the presence of at least one of the Pom-1 derivatives improved the viability reduction of fluconazole from 3 to 10 isolates of 28. The main observation that the resistant strains were included in the affected group of isolates also holds true for the fluconazole peptide combinations (Fig. 8B and S3B). In summary, the otherwise most effective 0.5x level left behind only four isolates which could not be eliminated either by the combination of peptides with fluconazole or amphotericin B, with isolate 23 surviving both regimes (Fig. 8).

**Fig. 9:**
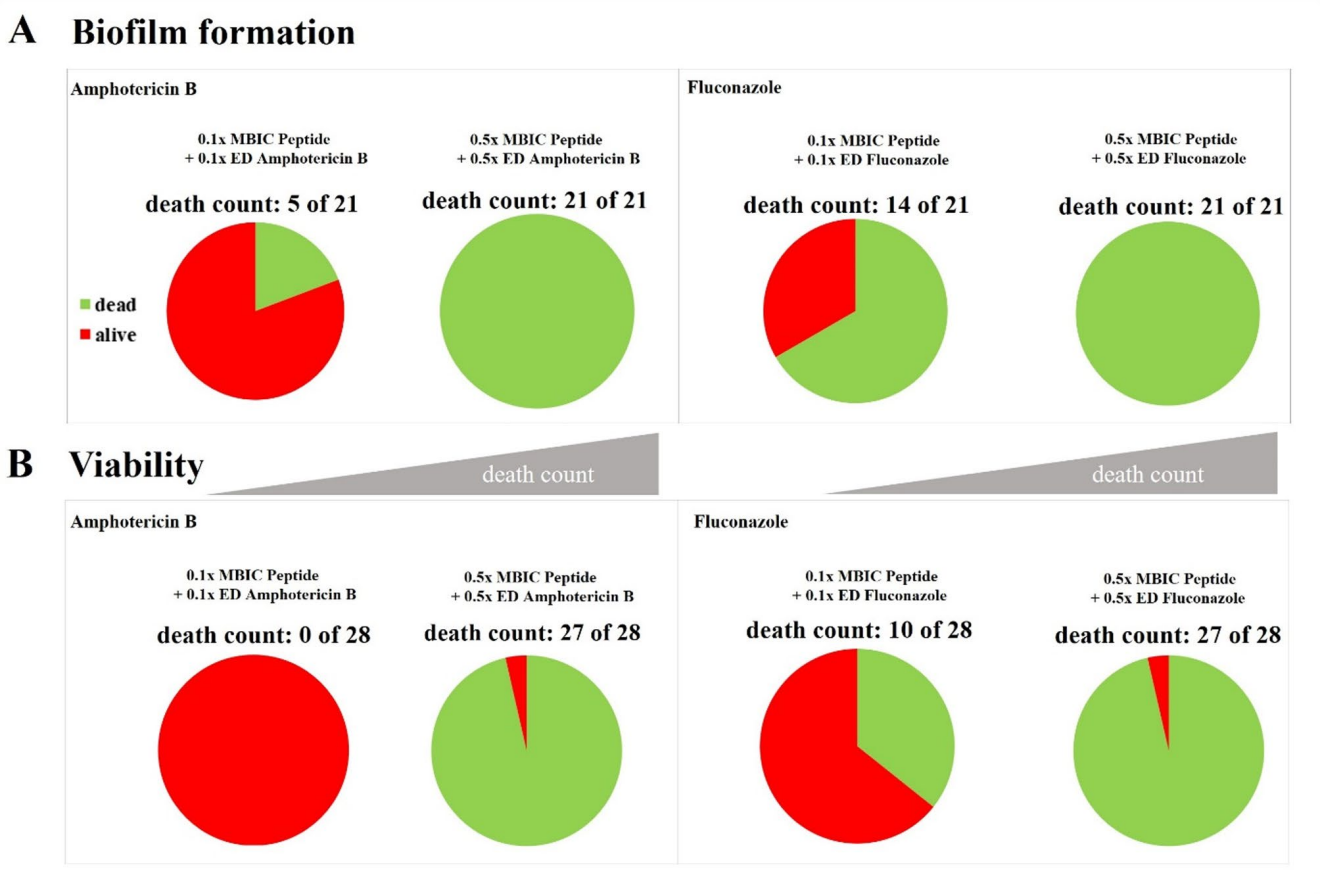
Further enhanced efficacy of standard antifungals in combination with all Pom-1 peptides in mixture against biofilm formation and cell viability. Pom-1 derivatives combined, as well as fluconazole and amphotericin B (0.1x MBIC peptides + 0.1x ED amphotericin B/fluconazole and 0.5x MBIC peptides + 0.5x ED amphotericin B/fluconazole. All experiments were performed in triplicate. (A) Biofilm formation of the *C. albicans* laboratory strain and clinical isolates after treatment with 0.1x MBIC of all Pom-1 derived peptides in mixtures combined with 0.1x ED amphotericin B (left panel) and fluconazole (right panel) as well as 0.5x MBIC peptides and 0.5x amphotericin B/fluconazole. (B) Cell viability of *C. albicans* laboratory strain and clinical isolates after treatment with 0.1x MBIC of all Pom-1 derived peptides in mixtures combined with 0.1x ED amphotericin B (left panel) and fluconazole (right panel) as well as 0.5x MBIC peptides and 0.5x amphotericin B/fluconazole.

Häring et al. presented our initial finding that the combination of all Pom-1 derivative peptides with fluconazole could enhance the peptides’ anti-biofilm activity at one specific submaximal effective concentration, which hence laid the foundation for this comprehensive study^33^. With the proviso to reduce the dose of fluconazole and/or amphotericin B to at least 50% of the normal effective dose, each of the Pom-1 derivatives alone did not succeed to reduce biofilm formation and cell viability for six and five of the clinical isolates, respectively (Figs. 7 and 8). To evaluate whether this combination strategy could be an option to reduce biofilm formation and viability of the remaining strains the Pom-1 peptides were used in mixtures but at the same final concentration as in the experiments presented before (0.1x MBIC and 0.5x MBIC) and again in combination with the same concentrations of the classical antifungals (0.1x ED and 0.5x ED). Whereas no effect was observed for the lower concentrations, at 0.5x MBIC and 0.5x ED biofilm formation of all strains was completely inhibited for both classical antifungals (Fig. 9A). The viability was reduced to zero by this combination for 27 of 28 isolates with only one single isolate surviving this combination of peptide mixtures with fluconazole or amphotericin B (Fig. 9B).

The isolate 23 was originally described to be impaired in the formation of regular biofilms in our biofilm assay on plastic surfaces^33^. Microscopic inspection of isolate 23 revealed that in addition the cells did not tend to aggregate or show regular hyphae (Figure S2A, untreated). Consequently, the application of Pom-1 (1x MBIC) did not result in obvious alterations of cell morphologies for this strain compared to the *C. albicans* control strain (Figure S2A, Pom-1). Moreover, isolate 23 was also unaffected by fluconazole and amphotericin B at1x ED whereas the lead peptide Pom-1 in combination improved the efficacy of amphotericin B and slightly for fluconazole, the derivatives delivered graded further improvements (Figure S2B). Although the derivative Pom-1E represented a significant improvement over the original Pom-1 peptide and the benchmark in this experiment with a reduction of viability to 25% in combination with both, fluconazole and amphotericin B, none of the individual peptides was suited to fully restore the efficacy of both traditional antimycotics and to completely eliminate viable cells. In this case the combination of all peptides in equal amounts reduced the peptide efficacy compared to the Pom-1E best performance suggesting that the *de facto* dilution of this peptide was critical in this experiment. Nevertheless, compared to the control experiments of this study using the traditional concentrations of 2 µg/mL amphotericin B and 8 µg/mL fluconazole (1x ED) which could not reduce viability to zero for 21 (amphotericin B) and 12 (fluconazole) of 28 strains, the combination strategy with the Pom-1 derivatives succeeded finally in 27 cases leaving only a single isolate behind. Remarkably, already 26 or 25 of the 28-member set were completely killed when at least one of the derivatives was present in only half of the traditional effective doses used in therapies with amphotericin B and fluconazole alone (0.5x ED), respectively. Without overestimating the exception of the highly resistant isolate 23, the concept of using Pom-1 derivatives as sensitizers to enhance and rescue efficacies of classical antifungals even at reduced concentrations succeeded considerably and elevated the ratio of completely killed *C. albicans* isolates from 57% for fluconazole and 25% for amphotericin B to a final value of dead cells of 96% for both traditional antimycotics, while meeting our intention to reduce their required concentrations.

## Discussion

The peptides Pom-1, and its designed derivatives Pom-1 A-F, have originally been isolated from the freshwater snail *P. poeyana* and were described as probable biofilm-dedicated antifungal compounds. They efficiently affected the establishment of elaborate biofilms of *C. albicans*, but this effect was less significant for other *Candida* species, and their influence on *C. albicans* cell viability was only marginal^28,34^. This led to the assumption that the Pom-1 peptides may possess a mode of action notably different from that of classical AMPs, which in most cases eliminate cells by the formation of pores^39–45^. It was suggested that the Pom-1 derivatives probably act on effector epitopes of the target cell, a mode of action that was also identified for certain antiviral peptides, which affect the viral membrane fusion peptide of, e.g. HIV^46^, and for the most therapeutic neutralizing antibodies^47–49^. *C. albicans* possesses a complex architecture cell surface with an external protein coat ordered in a mosaic-like structure, containing about 20 different protein species^50^. These cell wall proteins (CWPs) are mainly glycosylphosphatidylinositol proteins and/or covalently linked to the cell wall polysaccharides. They contribute to cell wall integrity, have been found to promote biofilm formation, mediate adherence to host cells and abiotic medical devices, promote invasion of epithelial layers, and offer protection against the immune system, thereby providing an important role in the fitness and virulence of *C. albicans*^51–54^. Agglutinin-like sequence proteins (Als) are prominent CWPs with known influence on biofilm formation and aggregation, which possess amyloid-forming propensity. In addition, CWPs like Csa1, Eap1, Hwp1, Pga10 and Rbt5 appear to have distinct functions in hyphae formation, aggregation and biofilm formation since upon loss of their deletion resulted in fragile biofilms or drastic reduction of biofilm formation^55–59^. Interestingly, Csa1p is not randomly distributed over the surface of yeast cells, but appears to be localized predominantly in the growing buds^55^. Thus it was suggested that the distribution of Csa1p may be restricted to sites of cell surface elongation^55^. Moreover, the N-terminal domain of Eap1 appeared to be responsible for yeast cell-cell adhesion and S/T-rich regions have been shown to mediate adhesion to abiotic polystyrene surfaces^56,57,60^. Here we focused on the anti-biofilm activity of the peptides during the early stages of biofilm development, applying the peptides directly at the start of cultivation to assess their ability to inhibit biofilm formation. We did not investigate their effects on mature/established biofilms or their potential to eradicate them, which remains to be investigated as prevention of biofilm formation is an important aspect of anti-biofilm strategies, but the ability to target and disrupt mature *C. albicans* biofilms remains a critical and particularly challenging goal, due to the high tolerance and complex architecture characteristic of these structures. To produce experimental evidence for both, the disability of Pom-1 to form pores and the idea that the Pom-1 peptides affect cell wall-related physiological functions like the formation of regular hyphae and cell-cell adhesion events in addition to their biofilm inhibiting activities, the microscopic analyses and the fluorescent permeabilization assay were performed. In essence, cells treated with the Pom-1 lead peptide displaying the expected alterations in aggregational behavior and morphology proved this hypothesis supported by the end-point measurements for the individual Pom-1 derivatives suggested that the normal functions of the putative peptide receptor epitopes were disengaged already in very early phases of cellular development in this kinetic assay. In consequence, the yet still questionable possibility of classical pore-formation activities could be excluded by the permeabilization assay. Whereas the known antimicrobial peptide control Cm-p5 from the marine mollusk *Cenchritis muricatus*^31^ as well as Triton X-100 mediated the uptake of fluorescent dyes into treated cells, Pom-1 treated cells remained completely unlabeled.

The introduction of classical natural AMPs in clinical trials faced several pharmacological challenges such as proteolytic digestion, short half-lives, increased levels of cytotoxicity upon systemic and oral application representing a crucial criterion of exclusion^37,61^. Although Pom-1 and its derivatives had been shown previously to be of very moderate cytotoxicity, which was also the case when they were applied in mixtures of the individual peptides, it was important to demonstrate that their combination with the aspired reduced doses of fluconazole and amphotericin B. According to the ISO norm ISO 10993-5 part 5 “Tests for in vitro cytotoxicity” a reduction of cell viability by more than 30% is considered a cytotoxic effect^37^. The relevant concentrations of peptides and classical antimycotics of 0.1x and 0.5x ED for the antifungals and MBIC for the peptides perfectly matched these criteria with toxicities not lower than 70% remaining viable cells. Considerable cytotoxicity was only detectable in the extreme concentration control containing 5x MBIC and 5x ED, and this was only for the combination of Pom-1 C with amphotericin B and solely for the A549 cell line. However, this exception appears to be not dramatic since A549 cells were chosen as a model because they can be regarded as ”hypersensitive” towards (classical) antimicrobial peptides, which in turn means that if a peptide is not harmful for A549 it is certainly not toxic for non-cancer cells^38^.

In summary, the key findings concerning efficacies against the formation of biofilms and, more importantly, for the reduction of metabolically active (planktonic) cells are that using half of the traditional concentrations (effective doses) of the established antimycotics fluconazole and amphotericin B were sufficient in combination with either one of the Pom-1 derivatives or in peptide mixtures to elevate the antifungal effects to a total of 96% killed cells (27 of 28 clinical isolates) and abolished biofilms (21 of 21 biofilm-forming isolates). Thus, the Pom-1 peptides remarkably fulfilled their intended function to boost the efficacies of fluconazole and amphotericin B and complied with the key requirement of our combination strategy to significantly reduce the burden of classical antifungal drugs during *C. albicans* treatment. The second envisioned aim of this concept is to use this boosting or rescuing effect to embank fluconazole and amphotericin B adverse effects into limitations which reduce harmful health consequences for the patients to more tolerable and less dangerous levels. Although this strategy appears to be promising in these aspects, it has to be stated that at the moment, it is completely unproven whether fluconazole and amphotericin B side effects decrease with their concentrations also in the presence of additional pharmaceutical compounds like the Pom-1 peptides. This has certainly to be considered also for all concepts for the synergistic use of additional compounds. Synergistic effects of these gold-standard antimycotics have been described for a range of microbial or plant-derived metabolites including Deferoxamine and gypenosides^62,63^. Moreover, although they show only marginal cytotoxicity, whether the individual Pom-1 derivative peptides or their mixture exhibit considerable adverse effects upon their use in animals and in the long-term in patients remains to be evaluated. Nevertheless, in our opinion, the unambiguous results presented here with only a limited collection of *C. albicans* strains isolated from invasive infections encourage us to proceed to these following developmental stages, including in-depth characterization of the combination strategy with larger sample numbers and more detailed immunological and toxicological evaluation also for the Pom-1 derivatives alone. Due to their strong anti-biofilm activity they may also serve as uniquely advantageous constituents of next-generation anti-*Candida albicans* preparations for so-called lock therapies as a conservative treatment option for catheter-related bloodstream infection in long-term catheterized patients^64^. We believe that the combination of the anti-biofilm, or anti-aggregation peptides, respectively, with classical antifungal drugs like fluconazole and/or amphotericin B may not only pave the way for innovative treatment strategies also against extremely resistant C. albicans per se but may also inspire improvements of peptide-loaded next-generation wound care materials which have already been developed as first defense lines against different pathogens including *C. auris*^65,66^.

## Materials and methods

### Materials

Acetic acid, agar-agar, crystal violet, 3-(N-morpholino)propanesulfonic acid (MOPS), peptone and yeast extract were acquired from Carl Roth GmbH (Karlsruhe, Germany). RPMI-1640 medium supplemented with L-glutamine was purchased from Thermo Fisher Scientific (Waltham, MA, USA) and resazurin sodium salt from Sigma-Aldrich Chemie GmbH (Steinheim, Germany). fluconazole was obtained from Merck KgaA (Darmstadt, Germany), and amphotericin B from Carl Roth GmbH (Karlsruhe, Germany). Propidium iodide was purchased from BD Biosciences (Franklin Lakes, NJ, USA), ATTO 488 alkyne from ATTO-TEC GmbH (Siegen, Germany), rhodamine phalloidin from Thermo Fisher Scientific (Waltham, MA, USA) and fluorescein (FITC) from Sigma-Aldrich Chemie GmbH (Steinheim, Germany). Each of Dulbecco’s modified Eagle’s medium (DMEM), fetal bovine serum (FBS) (10% (*w*/*v*)), and penicillin–streptomycin (100 U/mL, 1% (*w*/*v*)), as well as Accutase^®^ and Eagle’s minimum essential medium non-essential amino acids (MEM NEAAs) were obtained from Life Technologies (Carlsbad, CA, USA), as well as phosphate-buffered saline (PBS).

### Cell culture

Adenocarcinomic human alveolar basal epithelial cells (A549) (purchased from the ATCC, Manassas, VA, USA, CRM-CCL-185)^67^ and human dermal fibroblasts (HDFs) (purchased from tebu-bio GmbH, Offenbach, Germany) were used^68^ for the experiments. Cultivation was conducted in DMEM with FBS (10% (*w*/*v*), 15% (*w*/*v*)), MEM NEAAs (1% (*w*/*v*)), and penicillin–streptomycin (100 U/mL, 1% (*w*/*v*)) at 37 °C in an incubator containing 5% CO_2_.

### Passaging adherent cell cultures

Prior to passaging, a suitable medium (DMEM supplemented with 10% (*w*/*v*) FBS for A549, DMEM supplemented with 15% (*w*/*v*) FBS for HDFs) was preheated to 37 °C. The medium was removed from the culture flask, and 3 mL of Accutase^®^ were added to the adherent cells. The cells with Accutase^®^ were incubated for 5–10 min until they acquired a round morphology. To ensure complete cell detachment, the culture flask was slapped against the back of the hand. The desired number of cells was aliquoted into a new culture flask containing the preheated medium. The cells were then incubated at 37 °C with 5% CO_2_.

### Cultivation of *Candida* spp.

*C. albicans* laboratory reference strain (ATCC 90028) was purchased from the IPK Medical Mycology Laboratory and *C. albicans* clinical isolates were provided from patient samples sent to the Microbiology Department for diagnostic purposes. Isolates were collected anonymously, and the accreditation number of the Microbiology Department is DIN EN ISO15189:2014 (DAkks). All strains were cultured on Sabouraud dextrose agar (40 g/L glucose, 10 g/L peptone, 20 g/L agar, pH 5.6) For suspension cultures, individual colonies were inoculated in test tubes in 5 mL of RPMI-1640 supplemented with L-glutamine and grown at 37 °C with orbital shaking at 150 rpm for 16 h.

### Viability assay for cell culture

To detect the viability of human cells, a resazurin reduction assay was performed. For this, 2 × 10^4^ cells per well of a 96-well plate were incubated in 200 µL DMEM with additives at 37 °C and 5% CO_2_. After removing the medium, 100 µL of the medium and 100 µL of a peptide/antifungal solution (2.5 µg/mL, 25 µg/mL) were added. After incubation for 24 h at 37 °C and 5% CO_2_, 20 µL of a resazurin solution (0.15 mg/mL) were added into each well, and the cells were incubated again for 24 h at 37 °C and 5% CO_2_. Fluorescence measurement (excitation wavelength—535 nm, emission wavelength—595 nm) of the converted resorufin was then performed using a Tecan Infinite F200 microplate reader (Tecan Group Ltd., Männedorf, Switzerland).

### Molecular dynamics simulations

In order to explore the ability of Pom-1 derivative peptides with the *C. albicans* membrane, seven membrane systems were modelled. In all cases the membrane was prepared using a lipid composition mimicking the *C. albicans* ones: palmitoyloleylphosphatidylcholine, palmitoyloleylphosphatidylethanolamine, palmitoyloleylphosphatidylserine, palmitoyloleylphosphatidylinositol, and ergosterol (POPC/POPE/POPS/POPI/Erg) in a ratio 59:21:3:4:13^69,70^. The membrane was generated using the input generator from the Web site of CHARMM-GUI (https://www.charmm-gui.org, accessed: 03.05.2023)^71–74^. On the other hand, the 3D coordinates for the Pom-1 derivatives were taken from previous work^33^. Peptide molecules were added to one side of the membrane with its center of mass (COM) at 30 Å from the COM of the membrane, mimicking in vitro experiments in which peptide molecules are initially added to the yeast culture. All simulations were performed using the NAMD 2.14 package^75^ with the CHARMM36 force field^76–78^. The TIP3P water model was used to generate explicit solvation conditions^79^ and Newton’s equations of motion were integrated using the Verlet (leapfrog) algorithm^80^. Periodic boundary conditions were applied in all directions, and the cutoff of short-range van der Waals interactions was 1.2 nm. The particle mesh Ewald method^81^ was applied to treat long-range electrostatic interactions, with a 1.2 nm real-space contribution cutoff for Coulombic interactions. A temperature of 310 K° and a pressure of 1 atm were maintained by the Langevin thermostat^82^ and barostat^83^ respectively. In all systems, the protonation states of peptides were assigned based on calculations at pH 7 and with 150 mM NaCl. The systems were equilibrated in two steps. In the first place, a 1000 steps minimization followed by 0.5 ns of equilibration with the protein constraint was performed to guide the system to the nearest local energy minimum in configuration space. Secondly, the peptide was released from the harmonic constraints and the whole system was further equilibrated by another 0.5 ns. After the equilibration process, all simulations were performed for 100 ns under an isothermal − isobaric (NPT) ensemble without any restraints.

### Permeabilization assay

To demonstrate that Pom-1 does not form pores into the *C. albicans* membrane, a permeabilization assay was carried out. For this, 2.5 × 10^4^ cells/mL of the *C. albicans* reference strain were incubated in 200 µL of RPMI-1640 medium supplemented with either Pom-1 or Cm-p5 at final concentrations of 2.5 µg/mL and 10 µg/mL and incubated for 2 h at 37 °C. For the positive control, 100 µL of a 0.2% (*w*/*v*) solution of Triton X-100 was added 10 min prior to the end of the 2 h incubation time and for the negative control, no agent was added (untreated). After incubation, the tubes were centrifuged at 11,000 ×g and the supernatant was discarded. Afterwards, the cells were washed with once with PBS and after addition of 5 µL dye and 195 µL PBS (final concentration of dye: 5 µL/mL) the cell suspension was centrifuged at 11,000 ×g for 2 min. The supernatant was discarded, and the cells were resuspended in a 4% (*w*/*v*) solution of paraformaldehyde and incubated for 10 min in order to fixate the cells. Subsequently the yeast cells were washed 3x with PBS, resuspended in 200 µL of PBS and pipetted into flat-bottomed polystyrene microtiter plates with 96 wells (Sarstedt AG and Co. KG, Nümbrecht, Germany) and fluorescence measurements were conducted at an excitation wavelength of 498 nm (FITC), 535 nm (propidium iodide), 500 nm (Atto488), and 540 nm (rhodamine phalloidin) and an emission of 517 nm (FITC), 617 nm (propidium iodide), 520 nm (Atto488), 565 nm (rhodamine phalloidin) with a Tecan infinite F200 microplate reader (Tecan Group Ltd., Männedorf, Switzerland). Additionally, microscopic images were taken at 630x magnification.

### Biofilm formation and crystal violet assay

In order to evaluate the antifungal effects of the Pom-1 derivatives combined with amphotericin B or fluconazole on biofilm formation, a crystal violet assay was performed^84,85^. For this, 2.5 × 10^4^ yeast cells/mL were incubated in 200 µL of RPMI-1640 medium supplemented with amphotericin B, fluconazole and Pom-1 to Pom-1 F in flat-bottomed polystyrene microtiter plates with 96 wells for 24 h at 37 °C without agitation. Subsequently, the planktonic phase was removed, the remaining cells attached to the wells were washed twice with 200 µL of demineralized water and exposed to 200 µL of 0.1% (*w*/*v*) crystal violet solution for 15 min. Afterwards, the solution was removed and the cells were washed again twice with 200 µL of demineralized water and the plates were left to dry for 24 h at 25 °C. To dissolve the stain, 200 µL of 30% acetic acid was added and transferred to a new plate after 15 min. The absorbance was measured at 560 nm using a Tecan Infinite F200 microplate reader (Tecan Group Ltd., Männedorf, Switzerland). By comparing the resulting data against the untreated controls, the efficacy of the agents could be assessed.

### Resazurin-reduction-assay/viability-assay

The viability of *C. albicans* cells was assessed following the guidelines of the Clinical and Laboratory Standards Institute (M27-A3)^86^. Accordingly, 2.5 × 10^4^ yeast cells/mL were cultured in 200 µL of RPMI-1640 medium in the presence of peptide (Pom-1 to Pom-1 F) and amphotericin B or fluconazole in different concentrations (0.1x ED/MBIC and 0.5x ED/MBIC) in flat-bottomed polystyrene microtiter plates with 96 wells (Sarstedt AG and Co. KG, Nümbrecht, Germany) at 37 °C with shaking at 900 rpm on an Eppendorf shaker. The quantification of viable yeast cells was carried out using a Resazurin-Reduction-Assay^86^. Accordingly, the cells were exposed to 20 µL of a 0.15 mg/mL resazurin solution for 2 h and subsequently the amount of converted resorufin was determined with fluorescence measurements at an excitation wavelength of 535 and an emission of 595 nm with a Tecan infinite F200 microplate reader (Tecan Group Ltd., Männedorf, Switzerland).

## Electronic supplementary material

Below is the link to the electronic supplementary material.

Supplementary Material 1

## Data Availability

Data is provided within the manuscript or supplementary information files.
