# Supplementary material for: Anti-biofilm peptides can rescue fluconazole and amphotericin B efficacies against Candida albicans
Source: Sci Rep. 2025 Jul 9;15:24593. doi: 10.1038/s41598-025-10315-4 (PMC12241370; doi:10.1038/s41598-025-10315-4)
Supplement: Supplementary file 1 — Supplementary Material 1 [file 41598_2025_10315_MOESM1_ESM.docx]

**Supplementary Information**

Scientific Reports

**Pom-1 anti-*Candida albicans*-biofilm peptides rescue efficacies of the antifungals fluconazole and amphotericin B**

**Supplementary Material**


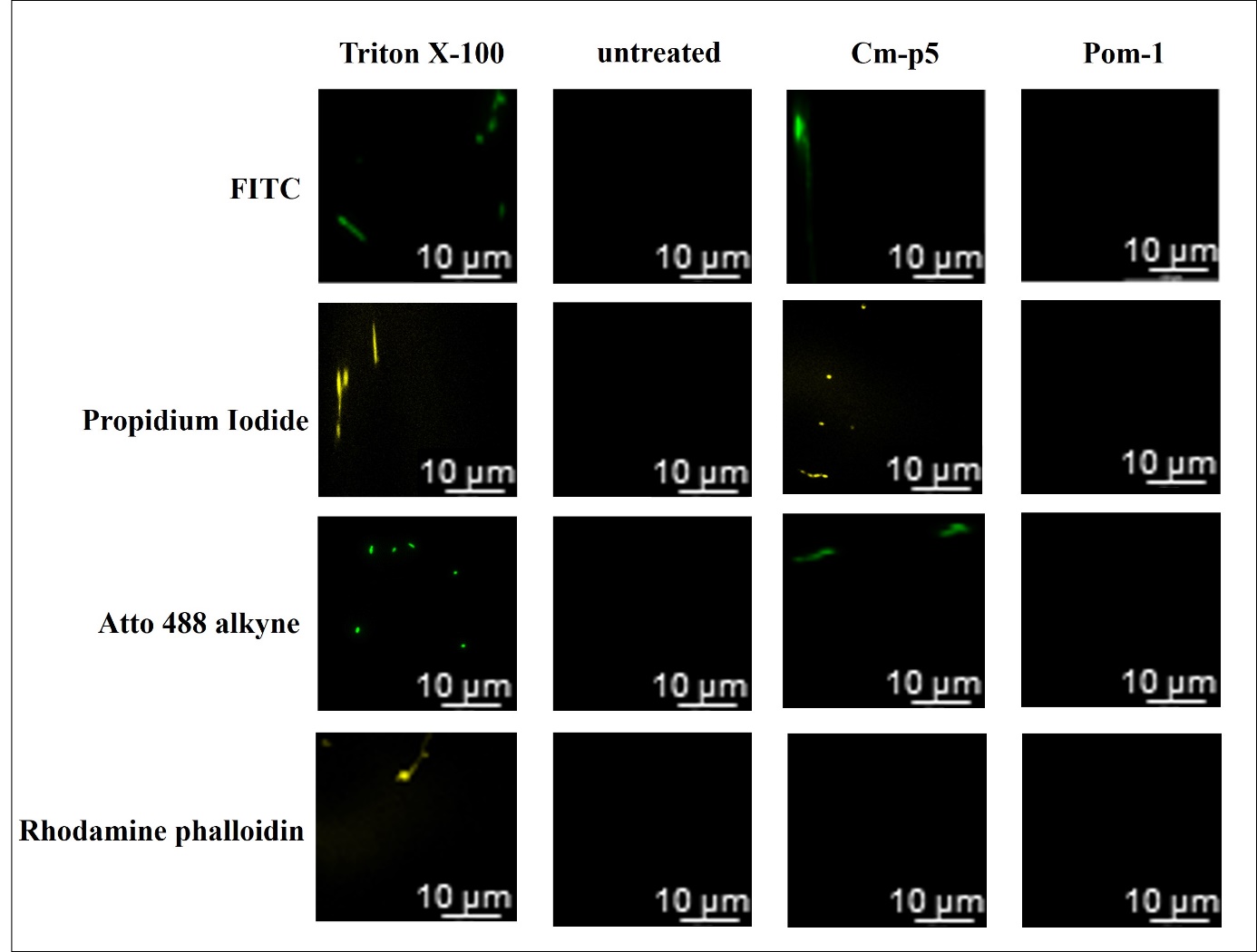


**Figure S1.** **Pom-1 has no influence on cell permeability whereas the known Cm-p5 increases the permeability of *C. albicans* cells. Fluorescence microscopy images were obtained after the permeabilization assay.** Shown are images of *C. albicans* cells after treatment with Triton X-100, Cm-p5, Pom-1 and untreated cells and consequent staining with the fluorescent dyes (increasing in size) FITC, propidium iodide, Atto 488 alkyne and rhodamine phalloidin.


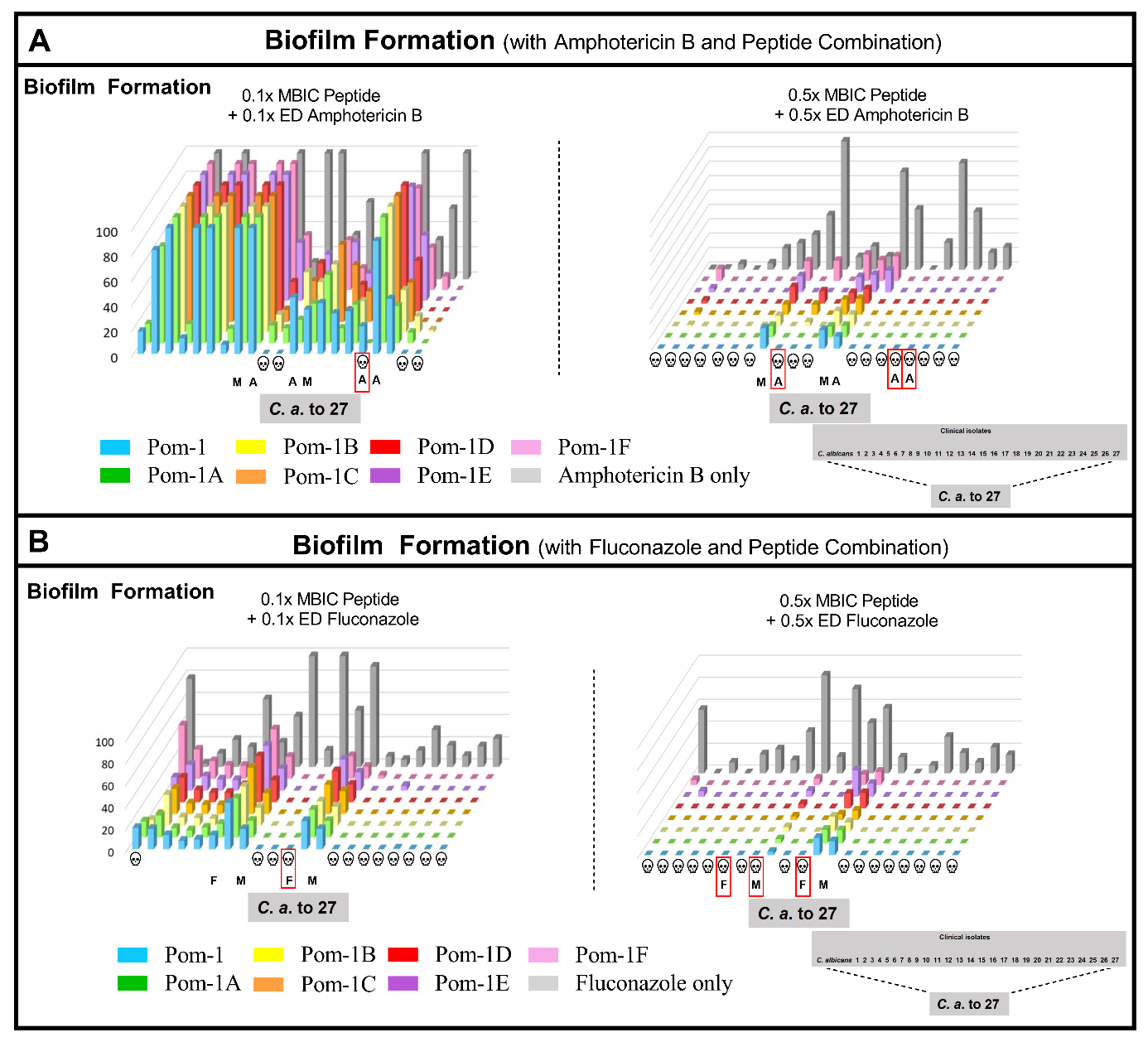


**Figure S2.** Biofilm formation of *C. albicans* clinical isolates detected by crystal violet assay after treatment with 0.1x MBIC peptide and A) 0.1x ED amphotericin B as well as 0.5x MBIC peptide and 0.5x ED amphotericin B and B) 0.1x ED with fluconazole B as well as 0.5x MBIC peptide and 0.5x ED fluconazole. Each bar represents one isolate, repeated for each Pom-1 derivative. The isolates were named according to their numbers and the laboratory strain as “*C. albicans*”. Each strain for that no biofilm formation could be detected for at least one of the Pom-1 derivatives was marked with a skull pictogram. Previously found amphotericin B resistant isolates are marked as “**A**”, fluconazole resistant isolates as “**F**” and multi-resistant isolates (resistant to amphotericin B and fluconazole) are marked as “**M**". All experiments were performed in triplicate.


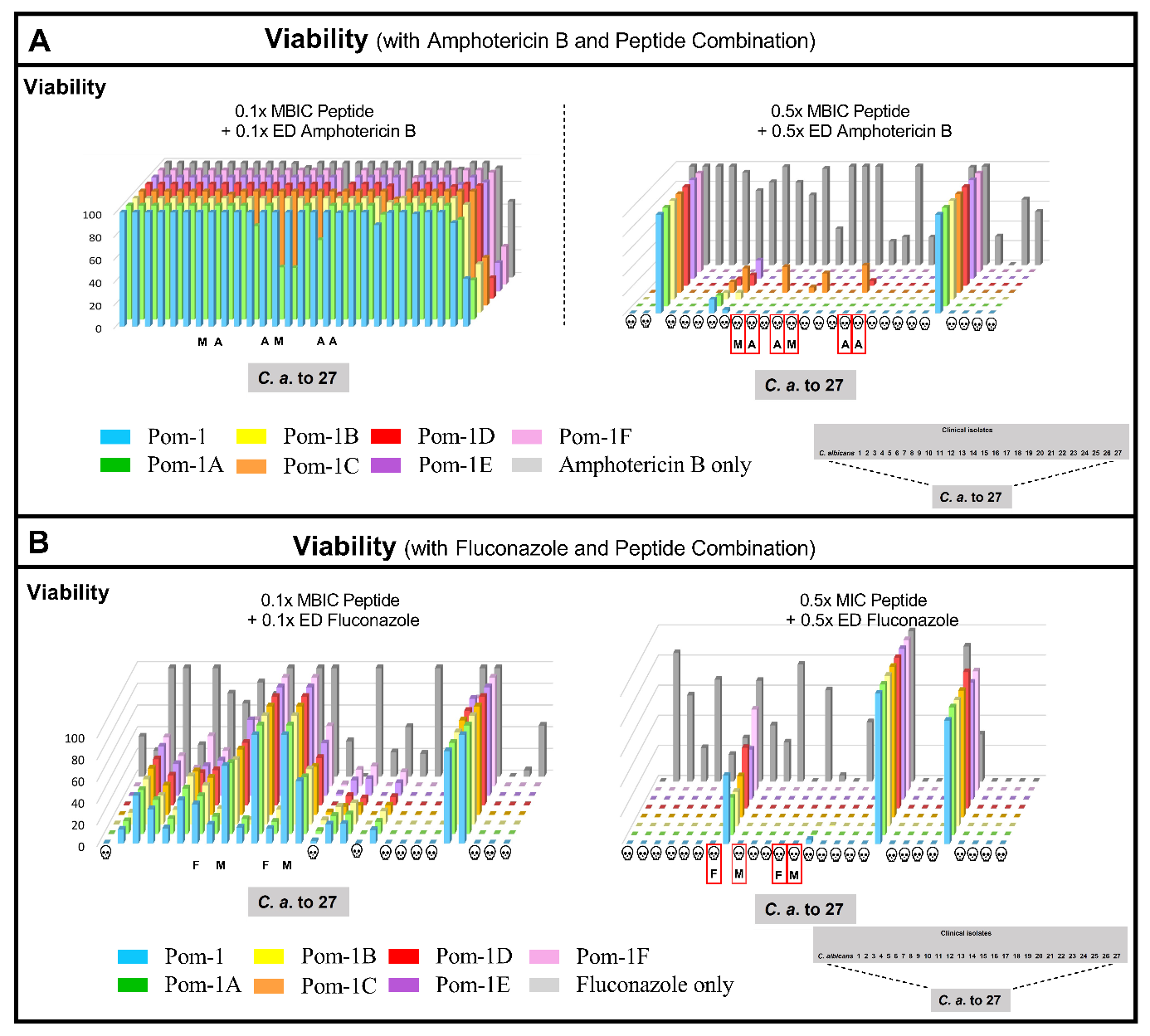


**Figure S3.** Viability of *C. albicans* clinical isolates detected by resazurin reduction assay after treatment with 0.1x MBIC peptide and 0.1x ED amphotericin B as well as 0.5x MBIC peptide and 0.5x ED amphotericin B or B) with 0.1x MBIC peptide and 0.1x ED fluconazole as well as 0.5x MBIC peptide and 0.5x ED fluconazole. Each bar represents one isolate, repeated for each Pom-1 derivative. The isolates were named according to their numbers and the laboratory strain as “*C. albicans*”. Each strain for that no cell viability could be detected for at least one of the Pom-1 derivatives was marked with a skull pictogram. Previously found amphotericin B resistant isolates are marked as “**A**”, fluconazole resistant isolates as “**F**” multi-resistant isolates (resistant to amphotericin B and fluconazole) are marked as “**M**". All experiments were performed in triplicate.


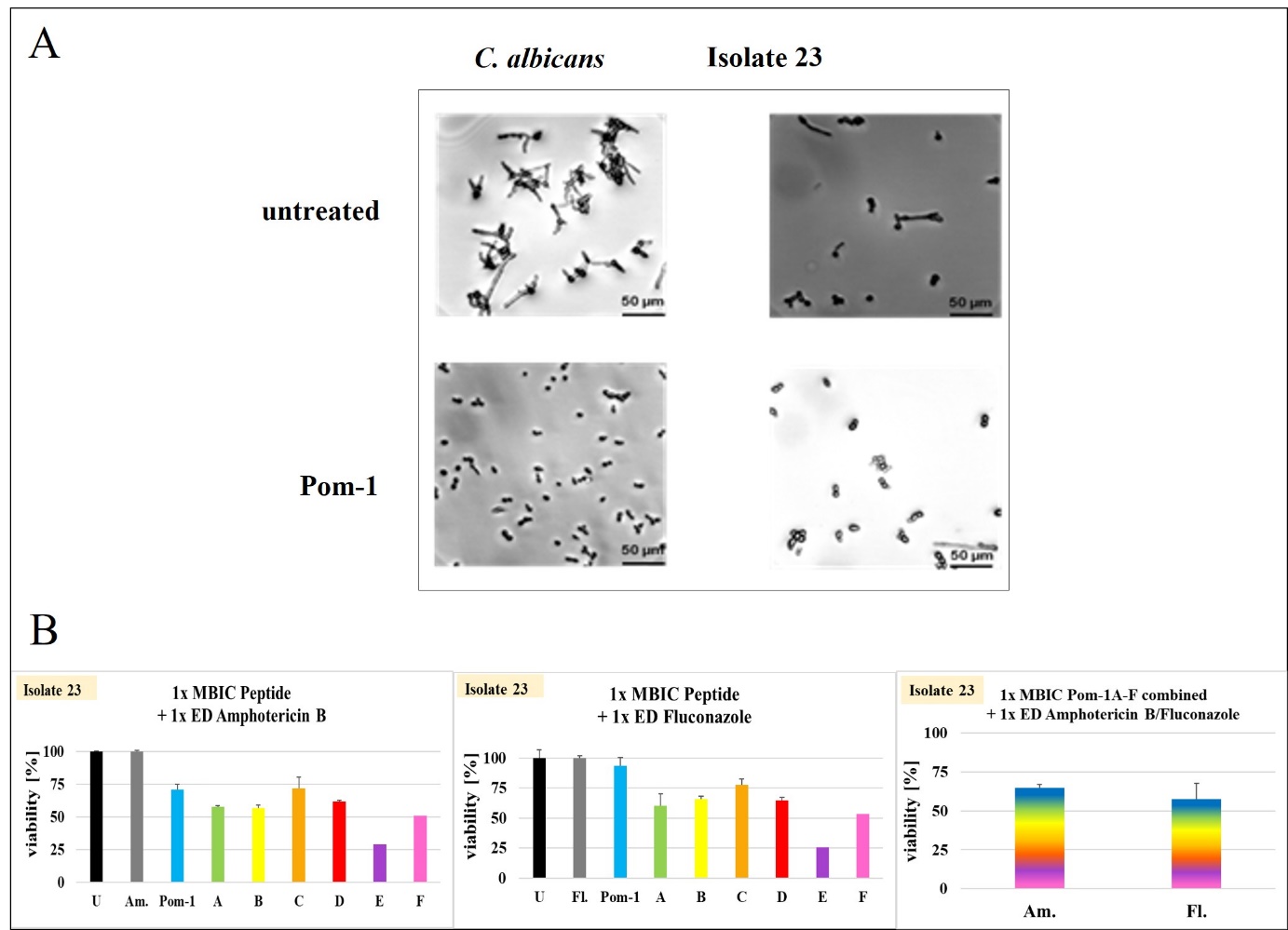


**Figure S4.** **Inherent resilience to aggregation by isolate 23 and marginal reduction of viability by further raised concentrations of antifungal and peptide combinations.** (A) Microscopic analysis using a Leica DMi8 coded (Leica Microsystems CMS GmbH, Wetzlar, Germany) microscope of *C. albicans* laboratory strain and clinical isolate 23 with and without treatment with Pom-1. The pictures were taken after an incubation time of 2 h. (B) Cell viability of clinical isolate 23 after treatment with 1x MBIC of the individual Pom-1 derivatives as well as 1x ED of amphotericin B/fluconazole and cell viability of isolate 23 after treatment with 1x MBIC of all Pom-1 derivatives in mixtures combined with 1x ED amphotericin B/fluconazole. “U” indicates the untreated control whereas “Am.” And “Fl.” represent amphotericin B and fluconazole. The individual Pom-1 derivatives are named according to their letter (A-F).
